# Supplementary material for: miR-130a and miR-145 reprogram Gr-1+CD11b+ myeloid cells and inhibit tumor metastasis through improved host immunity
Source: Nat Commun. 2018 Jul 4;9:2611. doi: 10.1038/s41467-018-05023-9 (PMC6031699; doi:10.1038/s41467-018-05023-9)
Supplement: Supplementary file 1 — Supplementary Information [file 41467_2018_5023_MOESM1_ESM.pdf]

miR-130a and miR-145 reprogram Gr-1+CD11b+ myeloid cells and inhibit tumor metastasis through improved anti-tumor immunity

Ishii et al.

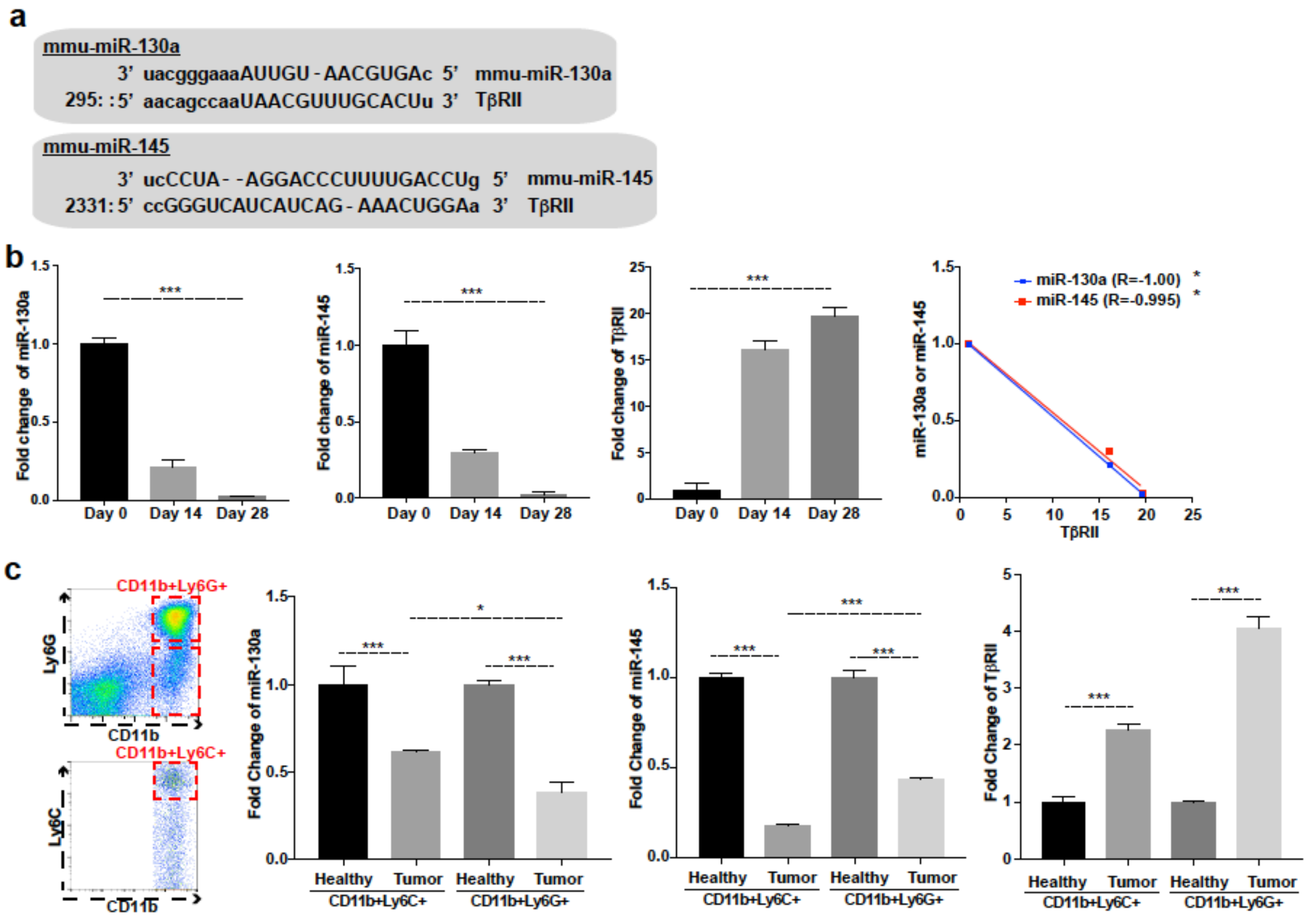

Supplementary Figure 1

(a) Seed sequences for miR-130a and miR-145 on TβRII 3'UTR (NM_009371). **(b)** qRT-PCR for fold changes of miR-130a, miR-145, or TβRII in sorted myeloid cells from the spleen of mice bearing E0771 tumors (orthotropic model) in a time course experiment. The correlations of miR-130a or miR-145 level with TβRII expression are on the right panel. Spearman analysis of the correlations of miR-130a or miR-145 level with TβRII level. R: Pearson's correlation coefficient. **(c)** Gating strategy of myeloid cell subsets (left). qRT-PCR for fold changes of miR-130a (left), miR-145 (middle) and TβRII (right) in sorted CD11b+Ly6C+ and CD11b+Ly6G+ cells from spleen of E0771 tumor-bearing mice (Tumor) compared with healthy control mice (Healthy). Data was represented as mean±SEM, and Student's *t* test was performed. *$p<0.05$, ***$p<0.001$.

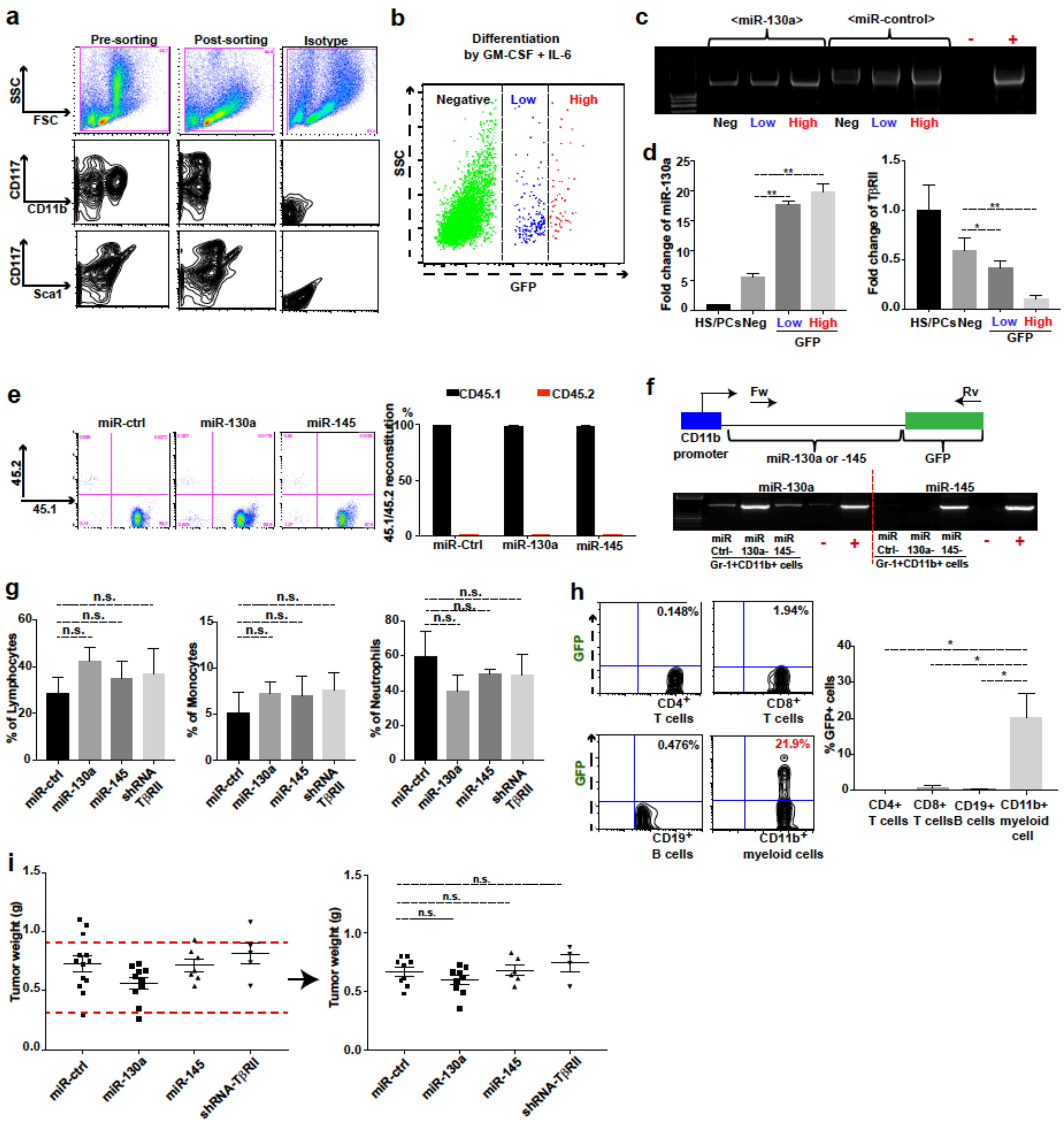

Supplementary Figure 2

(a) Cell sorting of enriched HS/PCs (Lin- population) used for lentivirus transduction experiments. **(b)** Flow cytometry of Gr-1+CD11b+ myeloid cells with different levels of GFP expression as indicated. **(c)** Electrophoresis after PCR amplification of DNA from sorted GFP-high, low, and negative cells, indicating the integration of the miR-130a expression vector in the genome of all populations. gDNA sample from HS/PCs was used as a negative control. **(d)** Expression of miR-130a (left) and TβRII (right) in myeloid cells compared to HS/PCs by qRT-PCR **(e)** Bone marrow reconstitution using CD45.1 and CD45.2 system, showing an efficient repopulation of donor bone marrow (CD45.1). Bone marrow reconstitution in transplanted mice was assessed by calculating the percentages of 45.1 (donor) vs 45.2 (recipient). **(f)** Genomic integration of miR-130a, miR-145 and control vector. Primers were designed as indicated by the arrows (upper panel). DNA-PCR was performed on the DNA extracted from Gr-1+CD11b+ myeloid cells sorted from the peripheral blood of the bone marrow transplanted mice. The samples were run on an agarose gel and the PCR product was shown (lower panel). **(g)** The leukocyte (lymphocytes,

monocytes, and neutrophils) counts from bone marrow transplanted mice at 10 wks after bone marrow transplantation. **(h)** Representative flow plots for GFP expression in T, B, and myeloid cells from the spleen of bone marrow transplanted mice (n=3). Quantitative data is on the right. **(i)** Tumor weight from mice received miR-130a–, miR-145–, or TβRII-shRNA–engineered bone marrow. The 4T1 cells were injected in the mammary fat pat number 2 and the tumor weights were assessed 28 days post injection. Mice with similar tumor weight were evaluated for lung metastasis numbers (left and right panels). Data was represented as mean±SEM, and Student's *t* test was performed. *p<0.05, **p<0.01.

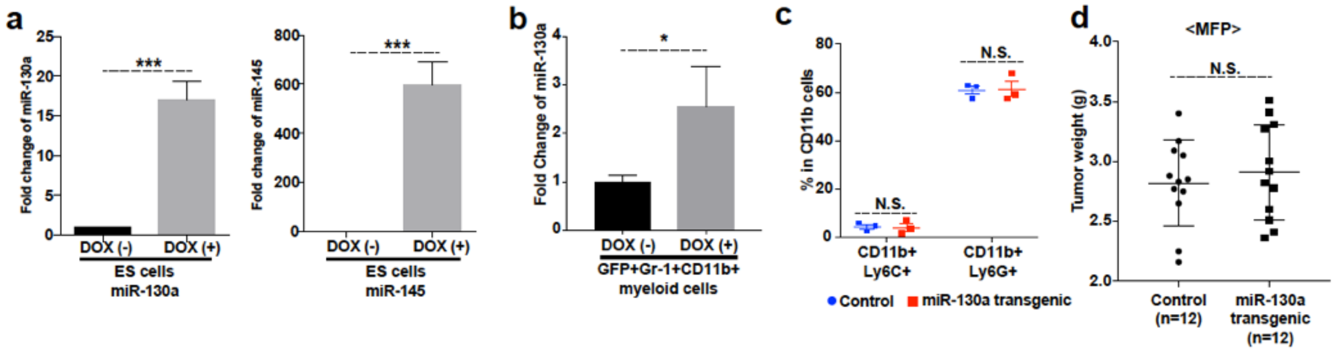

Supplementary Figure 3

(a) qRT-PCR: Tet-inducible expression of miR-130a and miR-145 in embryonic stem (ES) cells used to generate transgenic mice. The relative levels of miR-130a are 15 times more compared to vector control. On the other hand, miR-145 is 500 times more in ES cells treated with 1 µg/ml doxycycline (DOX) compared to untreated ES cells. **(b)** Tet-inducible miR-130a expression in sorted GFP positive Gr-1+CD11b+ myeloid cells from the spleen of DOX-treated miR-130a transgenic mice. **(c)** No difference in myeloid cell subsets from spleen between the miR-130a transgenic mice and Wt control mice when treated with DOX (n=3). **(d)** No difference in E0771 tumor weight between the miR-130a transgenic mice and Wt control mice when treated with DOX (n=12). Data was represented as mean±SEM and Student's *t* test was performed. *p<0.05, ***p<0.001, N.S.:not significant.

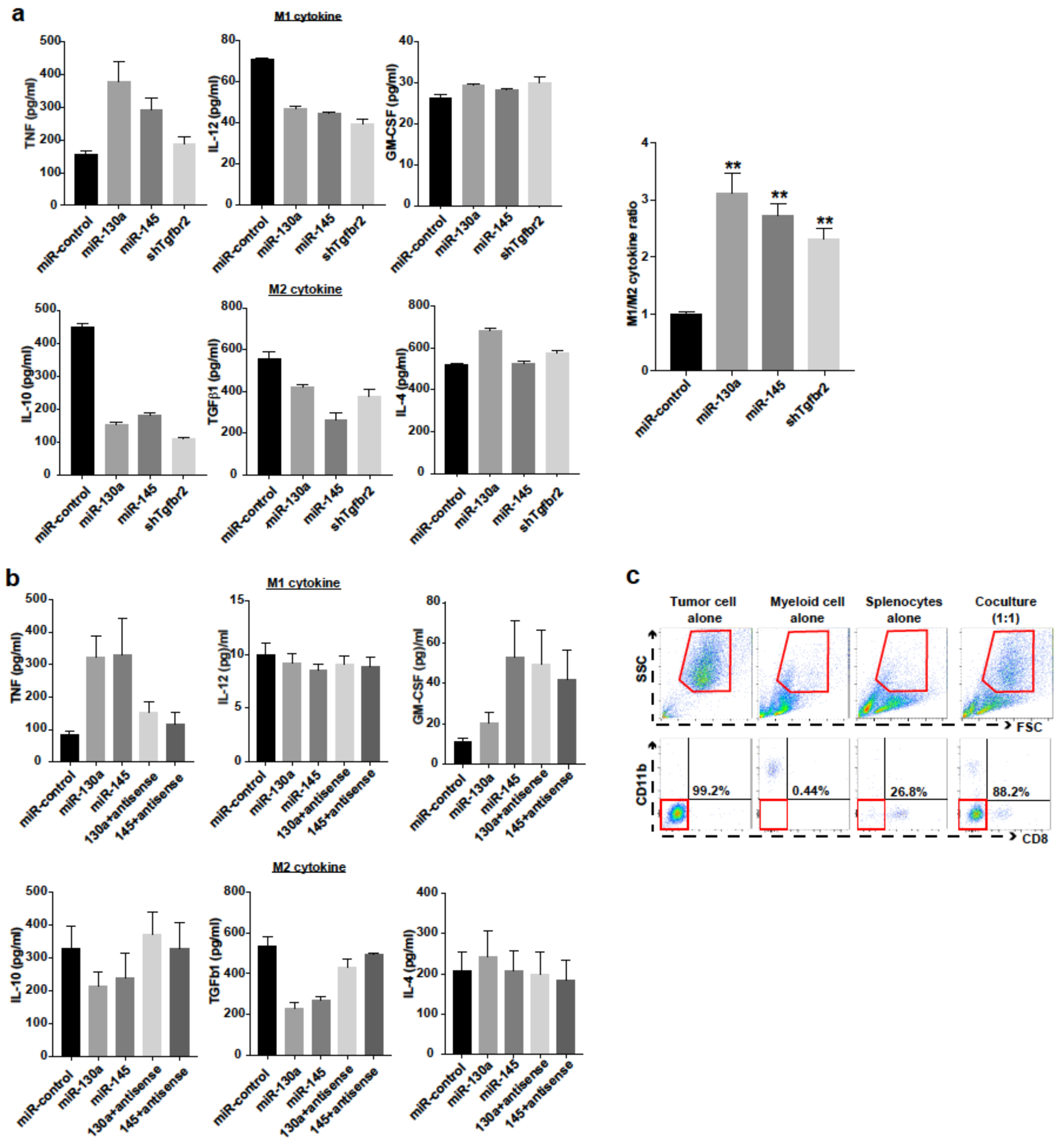

Supplementary Figure 4

(a) Ratio of M1 cytokines vs M2 cytokines in the culture supernatant of myeloid cells from 4T1 tumor-bearing mice that received miR-130a–, miR-145–, or TβRII-shRNA–engineered bone marrow. Cytokine expression was measured by Bioplex and ELISA, the ratio was calculated by dividing each M1 cytokine to M2 cytokine as described in Material and Methods. **(b)** Individual cytokine production in the culture supernatant of myeloid cells after treatment with miR electroporation with or without miR-130a or miR-145 antisense inhibitors (n=5). **(c)** Gating strategy of B16 melanoma cells expressing hgp100 after co-culture with splenocytes from Pmel-1 mice and sorted CD11b+ myeloid cells from the spleen of miR-130 transgenic mice for 24 hr. Data was represented as mean±SEM and Student's *t* test was performed. **$p<0.01$

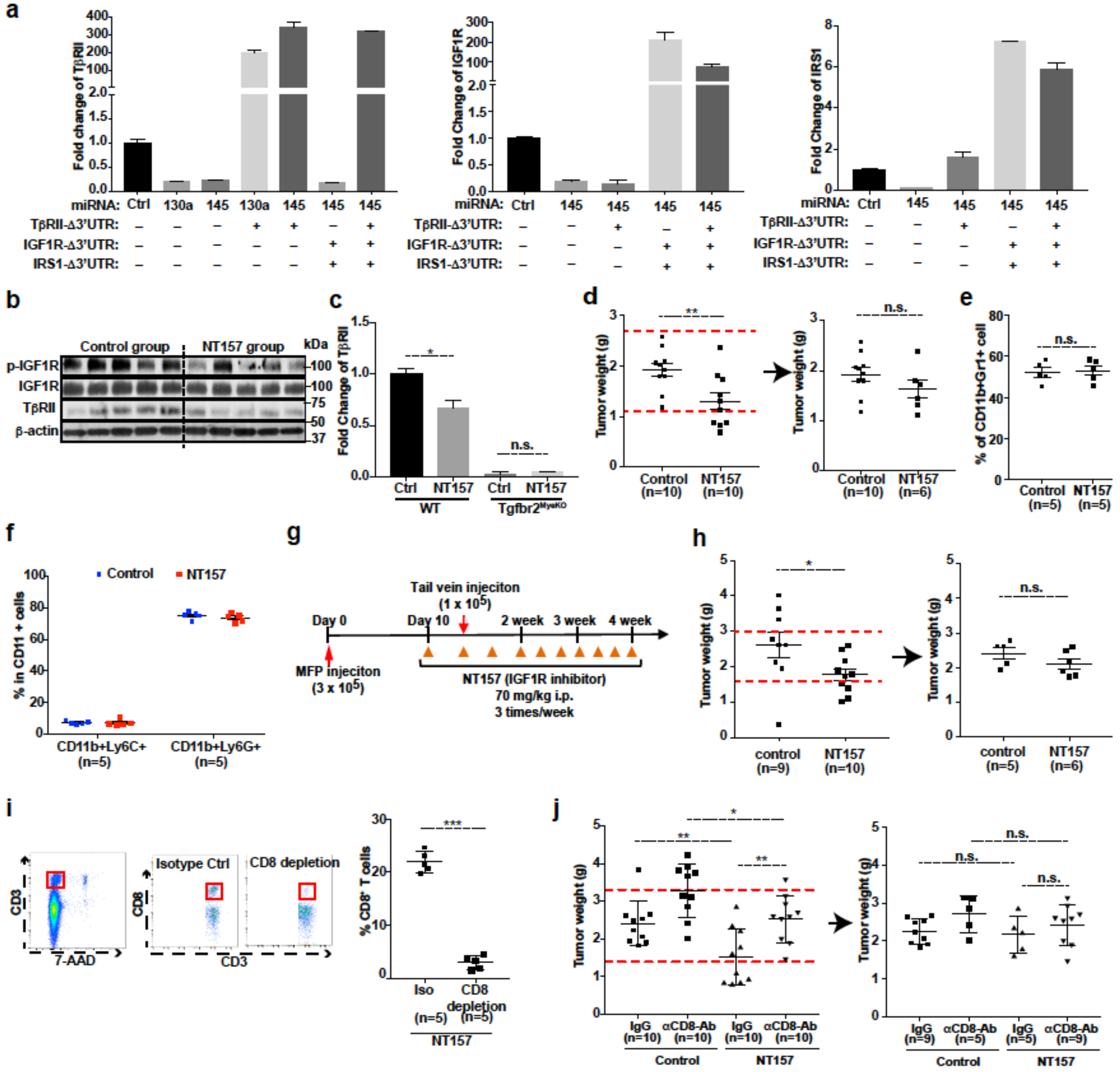

Supplementary Figure 5

(a) qRT-PCR of TβRII (left), IGF1R(middle), and IRS1(right) post restoration of TβRII, IGF1R, and IRS1 in Gr1+CD11b+ myeloid cells that overexpress miR-130a and miR-145. **(b)** Western blot of Gr-1+CD11b+ myeloid cells collected from spleen of 4T1 tumor-bearing mice treated with NT157 (n=5). **(c)** Expression of TβRII in Gr1+CD11b+ myeloid cells from spleen of WT or $Tgfbr2^{MyeKO}$ mice with NT157 treatment. **(d)** Tumor weight from 4T1 tumor-bearing mice treated with NT157 (n=10). Mice with similar tumor weight were evaluated for lung metastasis numbers (right panels). **(e)** The frequency of Gr-1+CD11b+ cell in spleen of 4T1 tumor-bearing mice treated with NT157 (n=5). **(f)** No effect of NT157 on myeloid cell subsets in spleen (n=5). **(g)** Schematic NT157 treatment for E0771 tumor-bearing mice. Mice received both MFP and tail vein injection as indicated. **(h)** Tumor weight from E0771 tumor-bearing mice treated with NT157 (n=9-10). Mice with similar tumor weight were evaluated for lung metastasis numbers (right panels). **(i)** Flow cytometry of CD8 T cells in mice treated with NT157, CD8+ T cell depletion, and IgG as control (n=5). **(j)** Tumor weight from E0771 tumor-bearing mice treated with NT157 and CD8+ T cell depletion (n=10). Mice with similar tumor weight were evaluated for lung metastasis numbers (right panels). Data was represented as mean±SEM and Student's *t* test was performed. *p<0.05,**p<0.01 ***p<0.001.

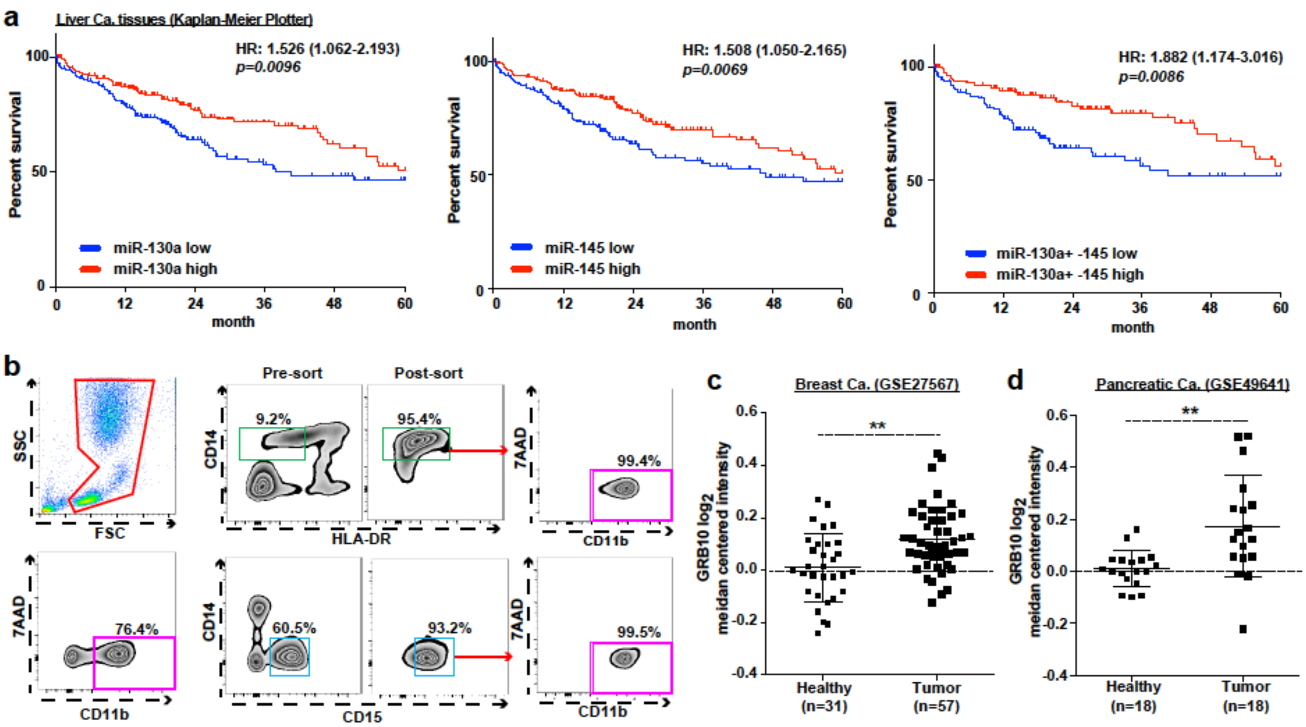

Supplementary Figure 6

(a) Kaplan-Meier survival curve (Kaplan-Meier Plotter) for liver cancer patients with high & low levels of miR-130a (left), miR-145 (middle), and both miR-130a and miR-145 (right) in tumor tissues. Log-rank test was used to analyze the survival data. HR: hazard ratio with its 95% confidence interval **(b)** Gating strategy of human myeloid cell subsest in flow cytemetry. **(c-d)** Increased GRB10 level in PBMCs from breast cancer patients(GSE27567) and pancreatic cancer patients (GSE49641) compared with healthy donors. Data was represented as mean±SEM, and Student's *t* test was performed. **p<0.01.

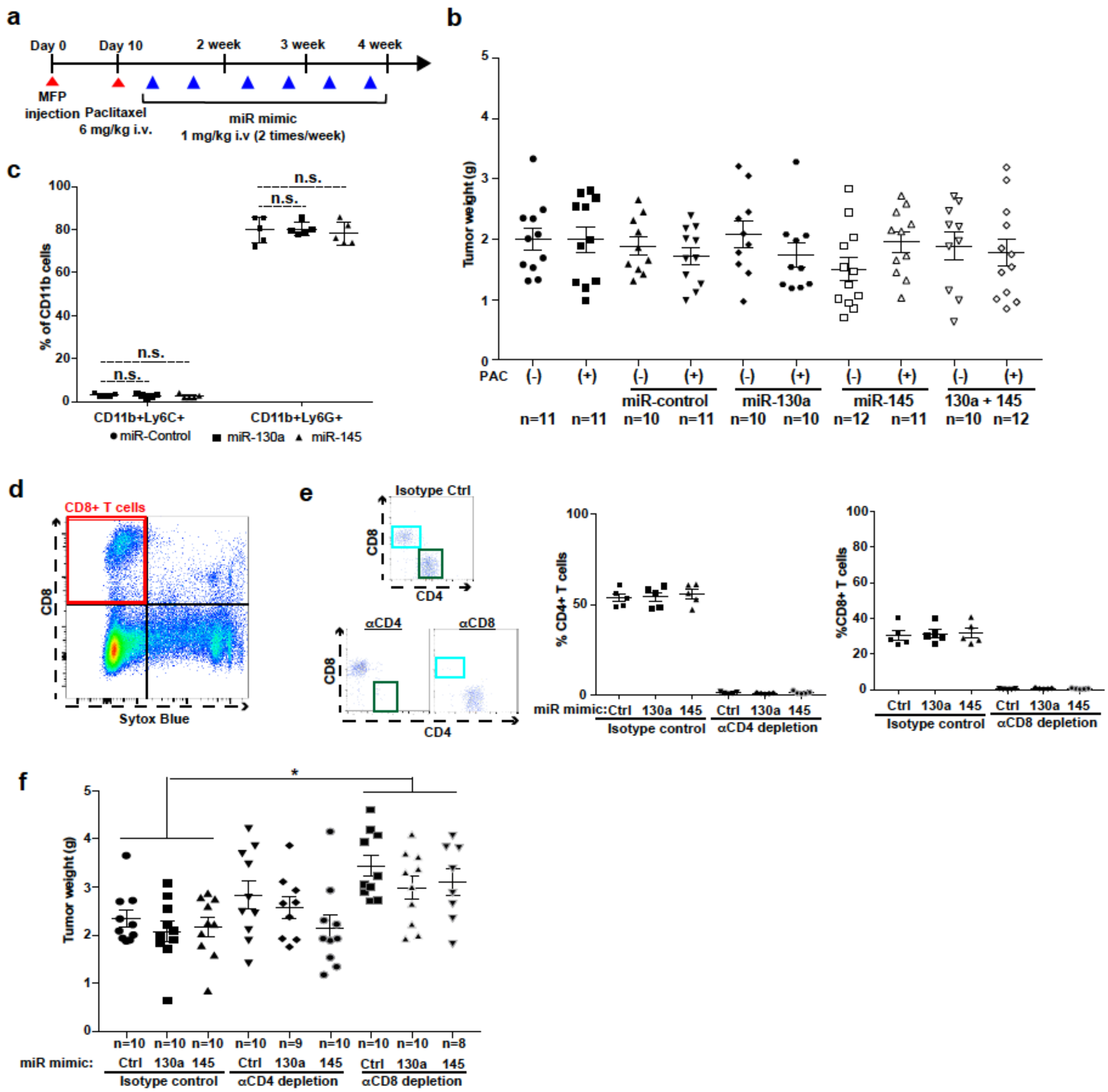

Supplementary Figure 7

(a) Schematic miR systemic delivery treatment with Paclitaxel for mice bearing 4T1 tumors. Mice received miR mimics and Paclitaxel by TVI as indicated. **(b)** Tumor weight from mice that received miR-control, miR-130a, and miR-145 mimics as single agent or in combination with Paclitaxel (6mg/kg) (n=10-12 mice). **(c)** No effect of systemic delivery of miR-mimics on myeloid cell subsets in the spleen (n=5). **(d)** Gating strategy of CD8+ T cells for T cell proliferation assay from the co-culture with myeloid cells from spleen of mice treated with miR mimics. **(e)** *In vivo* CD4+ or CD8+ T cell depletion after CD4 or CD8α neutralizing antibody and IgG control in 4T1 tumor-bearing mice treated with miR mimic injections (n=5). **(f)** Tumor weight after CD4+ or CD8+ T cell depletion in 4T1 tumor-bearing mice treated with miR mimic injections (n=8-10). Data was represented as mean±SEM, and Student's *t* test was performed. *p<0.05 **p<0.01, ***p<0.001

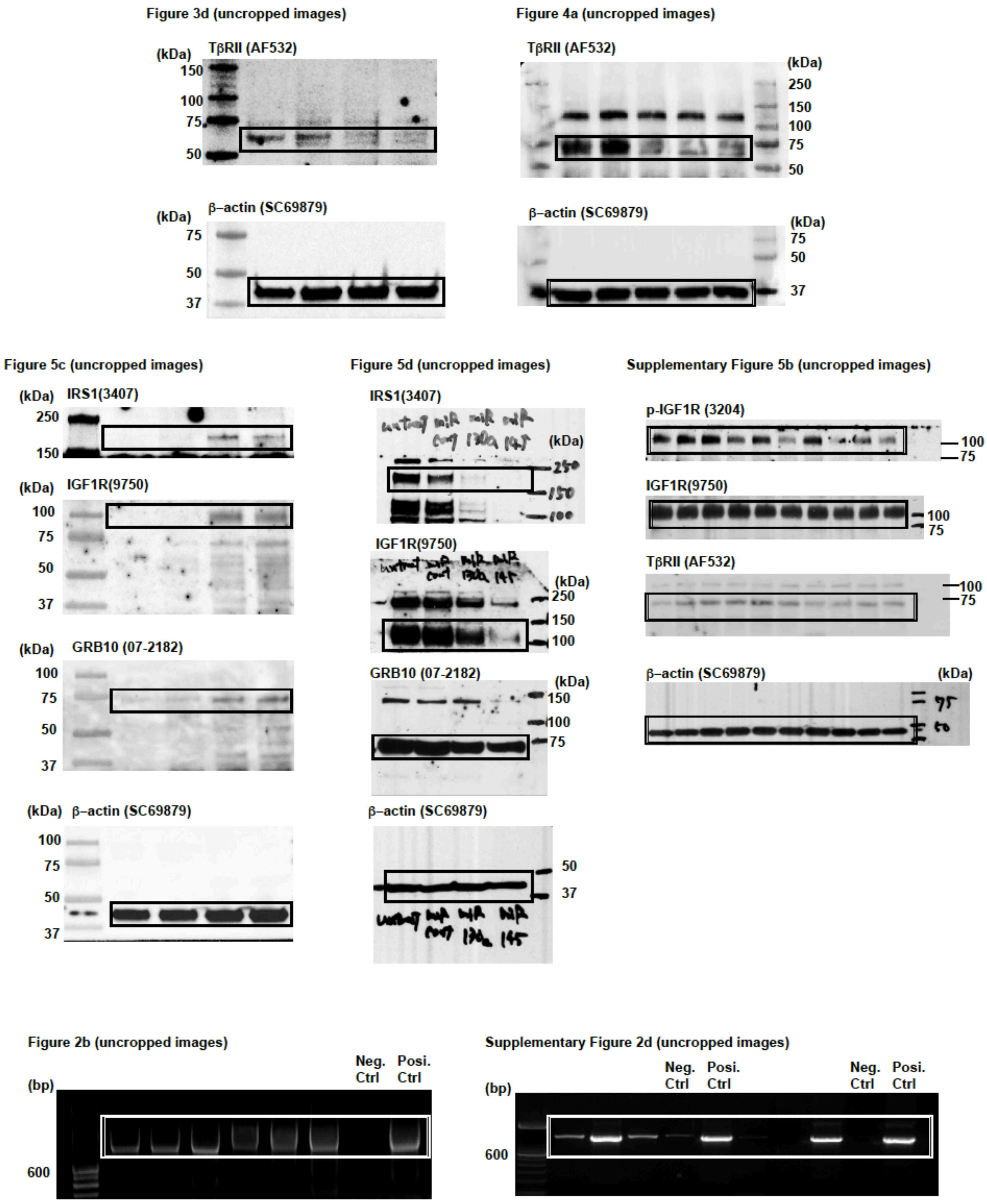

Supplementary Figure 8

Uncropped blots and gel images of figures presented in the main data and supplementary data.

Supplementary Table 1:

List of differentially expressed miRNAs in Gr-1+CD11b+ myeloid cells under tumor condition

	miR ID	Healthy condition			Tumor condition		
		sample 1	Sample 2	Sample 3	sample 1	Sample 2	Sample 3
Increased miRs	mmu-miR-582-5p	1	1	1	46	66	70
	mmu-miR-221	1	1	1	39	59	24
	mmu-miR-18a	1	1	1	20	24	12
	mmu-miR-222	1	1	1	13	24	13
	mmu-miR-1902	1	1	9	35	56	37
	mmu-miR-362-5p	1	1	1	15	15	1
	mmu-miR-503	1	1	1	14	7	1
	mmu-miR-1900	1	9	1	13	24	40
	mmu-miR-146b	1	1	1	1	8	11
	mmu-miR-532-3p	1	1	2	1	24	3
	mmu-miR-450a-5p	36	87	127	343	404	384
	mmu-miR-361	1	34	55	128	125	106
	mmu-miR-1198	30	32	33	123	117	132
	mmu-miR-340-3p	4	35	38	82	115	88
	mmu-miR-19a	42	65	139	264	292	298
	mmu-miR-223	14037	13049	13004	38074	41342	46966
	mmu-miR-139-5p	275	248	209	618	720	724
	mmu-miR-466a-3p+mmu-miR-466b-3-3p	1	7	26	20	47	31
	mmu-miR-148b	23	39	55	75	121	115
	mmu-miR-24	78	93	113	215	244	279
	mmu-miR-2134	97	133	51	344	183	191
	mmu-miR-423-5p	23	64	55	116	120	125
	mmu-miR-106b	100	106	123	225	322	280
	mmu-miR-29c	345	375	407	857	971	813
	mmu-miR-93	262	237	337	598	701	649
	mmu-miR-345-5p	17	5	4	7	29	24
	mmu-miR-423-3p	52	46	81	86	162	142
	mmu-miR-340-5p	702	540	647	1285	1414	1365
	mmu-miR-28	30	32	26	58	70	60
	mmu-miR-322	62	72	70	128	192	114
	mmu-miR-338-3p	46	29	50	101	90	76
	mmu-miR-2146	109	162	75	364	174	194
	mmu-miR-15b	4027	4082	4013	7649	9350	8422
	mmu-miR-19b	192	93	209	314	396	301
	mmu-miR-16	8624	10504	10225	17779	21138	19308
	mmu-miR-148a	266	261	269	489	616	471
	mmu-miR-21	3523	2994	3724	6481	6398	7100
Decreased miRs	mmu-miR-181b+mmu-miR-181d	33	13	14	1	12	19
	mmu-let-7f	501	325	323	172	154	252
	mmu-miR-674	4	1	1	1	1	1
	mcmv-miR-m108-1	4	1	1	1	1	1
	mmu-miR-770-3p	4	1	1	1	1	1
	mmu-miR-1965	4	1	1	1	1	1
	mmu-miR-672	4	1	1	1	1	1
	mmu-miR-682	4	1	1	1	1	1
	mmu-miR-30e	74	91	124	44	39	53
	mghv-miR-M1-5	65	20	9	6	9	27
	mmu-miR-126-3p	231	196	193	165	33	57
	mmu-miR-2135	138	59	71	57	20	18
	mmu-miR-181a	1506	991	880	380	350	414
	mmu-let-7b	600	629	589	194	180	217
	mmu-miR-200b	106	56	33	14	17	31
	mmu-miR-2137	52	26	15	13	7	8
	mmu-miR-142-5p	106	20	20	1	8	33
	mmu-miR-30c	68	28	36	1	26	11
	mmu-miR-709	119	65	74	26	10	25

	mmu-miR-146a	1222	1162	1067	254	242	318
	mmu-miR-150	6941	6944	5795	1071	951	1916
	mmu-miR-451	208	130	154	60	12	21
	mmu-miR-30a	93	69	50	14	15	8
	mmu-miR-145	42	64	56	13	9	4
	mmu-miR-135a	11	14	16	1	4	1
	mmu-miR-133a	62	3	13	1	1	10
	mmu-miR-467f	20	21	16	1	6	1
	mmu-miR-714	7	15	2	1	1	1
	mmu-miR-130a	1	37	54	7	1	1
	mmu-miR-181c	30	1	6	1	1	1
	mmu-miR-155	84	68	80	8	1	8
	mmu-miR-342-3p	253	231	179	1	11	24
	mmu-miR-99a	11	31	17	1	1	1
	mmu-miR-152	7	17	35	1	1	1
	mmu-miR-151-3p	17	37	25	1	1	1
	mmu-miR-804	30	35	29	1	1	1
	mmu-miR-125b-5p	46	32	56	1	1	1
	mmu-miR-151-5p	109	85	73	1	1	1

Supplementary Table 2: List of genes targeted by miR130a and miR145

Common targeted genes	miR130a targeted genes		miR145 targeted genes	
Abca1	Aak1	Nus1	Actg1	Map3k1
Cfl2	Abcb7	Pcgf5	Adcyap1	Mbtd1
Grb10	Ankib1	Peli1	Akap12	Mdfi
Kcna4	Apcdd1	Plaa	Angpt2	Nfe2l1
Pvrl3	Atg14	Psd	Ap2b1	Nr4a2
Tgfbr2	Atp6v1b2	Rc3h2	Ap3s2	Otud7b
Zfpm2	Atxn1	Rnf2	Arih1	Pafah1b2
	Bcl2l11	S1pr1	Cachd1	Pax3
	Birc6	Sash1	Camk2d	Pkn2
	Blcap	Slc44a1	Celf2	Plagl1
	Ccdc88a	Slc8a1	Clcn3	Plekhh1
	Cdk5rap3	Smad4	Col4a3	Rbpms
	Clcn5	Smoc2	Crkl	Rgs7
	Cnot7	Snx5	CTNND1	Rrp7a
	Cxcl12	Socs2	Dab2	Rtkn
	Dynll2	St8sia3	Derl2	Scamp3
	Eda	Sub1	Dlx6	Serpine1
	Elk3	Tardbp	Dusp6	Slc4a1ap
	Enah	Tbcel	Efnb3	Smad1
	Eps15	Tex261	Eif4a2	Smad3
	Fermt2	Timp2	Eif4ebp2	Smarca5
	Fosl2	Tnpo1	Ext1	St13
	Fubp1	Trove2	Fam60a	Timm23
	Fyn	Uba3	Flt1	Tln1
	Grik2	Ubap2l	Fzd4	Tpm4
	Hoxb3	Ucp3	Gdnf	Trio
	Limd2	Vps37b	Gosr2	Tspan6
	M6pr	Wee1	Gphn	Ulk1
	Mapk8	Wnk1	Gtf3c4	Vamp4
	Mdm4	Wnt1	Igf1r	Wdfy2
	Mybl1	Zfp131	Irs1	Yes1
	Myh14	Zfp91	Kif3a	Znrf2
	Myt1l	Zmat3	Leng8	
	Nhsl2			

Supplementary Table 3: Primer sequence for conventional PCR and qRT-PCR

	Forward primer sequence	Reverse primer sequence
**miR ctrl-gfp vector**	5'-TGATAGACTTCTGCCTCCTACT-3'	5'-AACGACACCTGAAATGGAAGA-3'
**miR130a-gfp vector**	5'-GTGCTACTGTCTAACGTGTACC-3'	5'-CCATGTGATCGCGCTTCT-3'
**miR145-gfp vector**	5'-GGAATCCCTTGGATGCTAAGAT-3'	5'-ACTGGGTGCTAGGTAGT-3'
**mouse Tgfbr2**	5'-AGTGATGTCATGGCCAGCGAC-3'	5'-CGCAGACTTCATGCGGCTTCTC-3'
**mouse Tgfbr2**	5'-TTTCTCCGTTGTGACTCGTG-3'	5'-GTGGCGAAAACCCAGACTTA-3'
**mouse Irs1**	5'-TGTGGCCACTCAGAGAACTT-3'	5'-GTCTCATGCATGTTCTGGGC-3'
**mouse Igf1r**	5'-GATGAGTGCATGCAGGAGTG-3'	5'-CTTCATCGCCGCAGACTT-3'
**mouse Arg1**	5'-GATTGGCAAGGTGATGGAAG-3'	5'-TCAGTCCCTGGCTTATGGTT-3'
**mouse Tgfb1**	5'-CTCCCGTGGCTTCTAGTGC-3'	5'-GCCTTAGTTTGGACAGGATCTG-3'
**mouse Il10**	5'-TTTGAATTCCCTGGGTGAGAA-3'	5'-GGAGAAATCGATGACAGCGC-3'
**18S rRNA**	5'-GTAACCCGTGAACCCCATT-3'	5'-CCATCCAATCGGTAGTAGCG-3'
**mouse Gapdh**	5'-AATGTGTCCGTCGTGGATCTGA-3'	5'-GATGCCTGCTTCACCACCTTCT-3'
