# Supplementary material for: miR-130a and miR-145 reprogram Gr-1+CD11b+ myeloid cells and inhibit tumor metastasis through improved host immunity
Source: Nat Commun. 2018 Jul 4;9:2611. doi: 10.1038/s41467-018-05023-9 (PMC6031699; doi:10.1038/s41467-018-05023-9)
Supplement: Supplementary file 3 — Description of Additional Supplementary Files [file 41467_2018_5023_MOESM3_ESM.pdf]

Description of Additional Supplementary Files

File Name: Supplementary Data 1
Description: List of differentially expressed genes in Gr-1+CD11b+ myeloid cells under tumor condition
